# Venous sinus stenting for pulsatile tinnitus secondary to venous sinus stenosis: a dual-center retrospective cohort study

**DOI:** 10.1007/s10143-026-04412-9

**Published:** 2026-07-24

**Authors:** Amr Abu Elfadle, Mohamed S. Hemeda, Mohamed Abd ELRhman Elassy, Wael Salah, Ahmed Nada, Mohamed Reda Abd El aziz Zaki, Mohammed Elghareeb

**Affiliations:** 1 Department of Neurosurgery, Faculty of Medicine, Port Said University, Port Said, Egypt; 2 Department of Forensic Medicine and Clinical Toxicology, Port Said University, Port Said, Egypt; 3 Department of Otolaryngology, Faculty of Medicine, Port Said University, Port Said, Egypt; 4 Department of Neurosurgery, Faculty of Medicine, Zagazig University, Zagazig, Egypt; 5 Department of Neurosurgery, Qena University Hospitals, Qena University, Qena, Egypt

**Keywords:** Pulsatile tinnitus, Venous sinus stenosis, Venous sinus stenting, Lumbar puncture opening pressure, Transverse sinus, Endovascular therapy

## Abstract

Pulsatile tinnitus (PT) can emerge due to stenosis in the venous sinus, which leads to turbulent venous flows and increased pressures within the veins. Stenting of the venous sinus (VSS) is a potential intervention to relieve PT and is considered minimally invasive, as it restores proper venous flow. To evaluate the clinical, hemodynamic, and radiological outcomes of venous sinus stenting (VSS) in a real-world cohort of patients with pulsatile tinnitus (PT) and venous sinus stenosis, and to describe the symptom–imaging–manometry framework used for procedural selection in routine practice. From January 2018 to December 2024, we conducted a dual-center, retrospective cohort study of 30 consecutive adults with PT who underwent transverse sinus stenting. Secondary outcomes included changes in trans-stenotic venous pressure gradient, early 1-month change in LP opening pressure, radiologic resolution, papilledema improvement, procedure-related complications, and recurrence. Thirty patients were included (28 females; mean age 39.0 ± 13.1 years). PT was right-sided in 50.0%, left-sided in 16.7%, and bilateral in 33.3%. Mean pre-stent trans-stenotic pressure gradient was 7.0 ± 1.4 mmHg and decreased significantly after stenting (P < 0.001). LP opening pressure decreased from 39.93 ± 10.99 before stenting to 18.23 ± 3.87 cm H₂O at 1 month after stenting (P < 0.001). Immediate complete symptom resolution occurred in 27 patients (90%); the remaining 3 patients (10%) achieved complete resolution by 12 months of follow-up. Follow-up MRV demonstrated resolution of the treated venous sinus stenosis, with restored sinus caliber/patency in all patients. Minor complications occurred in 3 patients (10%) (ecchymosis 6.7%, epistaxis 3.3%), with no major adverse events or recurrence. VSS was associated with a significant reduction in venous pressure gradient and LP opening pressure, along with high durable PT resolution rates and a low rate of minor complications in this cohort.

**Supplementary Information:**

The online version contains supplementary material available at 10.1007/s10143-026-04412-9.

## Introduction

Pulsatile tinnitus (PT) is a pulse-synchronous auditory perception that often reflects an underlying vascular or hemodynamic source rather than primary cochlear dysfunction. Because many causes of PT are identifiable and potentially treatable, clinical assessment and targeted vascular imaging are essential for diagnosis and treatment planning [1, 2]. Among venous causes, transverse or sigmoid sinus stenosis, sigmoid sinus wall abnormalities including diverticulum, prominent venous channels, and abnormal venous outflow may generate turbulent venous flow and produce pulse-synchronous symptoms [3–8].

Venous sinus stenting (VSS) is a minimally invasive endovascular treatment option for carefully selected patients with venous PT and hemodynamically significant venous sinus stenosis. By restoring venous sinus caliber and reducing the trans-stenotic pressure gradient, VSS may relieve tinnitus and associated symptoms. Previous series have reported favorable outcomes in both isolated PT and PT associated with idiopathic intracranial hypertension (IIH) or raised intracranial pressure physiology [9, 10]. However, the literature remains heterogeneous with respect to patient selection, manometric thresholds, outcome definitions, and follow-up methodology [11–13]. In retrospective practice, formal IIH classification may also be limited when ophthalmologic findings and other diagnostic elements are not uniformly documented.

Therefore, this study aimed to evaluate the clinical, hemodynamic, physiologic, radiologic, and safety outcomes of VSS in a consecutive dual-center real-world cohort of patients with PT secondary to venous sinus stenosis. The main contribution of this study is the combined reporting of symptom resolution, trans-stenotic pressure gradient change, radiologic resolution, papilledema improvement, and procedure-related complications within a symptom–imaging–manometry selection framework used in routine practice. The 1-month LP opening pressure change was analyzed as a supportive secondary early physiologic outcome.

## Materials and methods

### Study design and setting

This is a dual-center retrospective cohort study conducted between January 2018 and December 2024 at Qena University Hospital and Port Said Comprehensive Health Insurance Hospital. The study is reported in accordance with the STROBE guidelines for observational studies.

## Results

A total of 30 patients with pulsatile tinnitus (PT) secondary to venous sinus stenosis underwent venous sinus stenting during the study period. The cohort was predominantly female (93.3%), with a mean age of 39.0 ± 13.1 years. PT laterality was right-sided in 50.0%, left-sided in 16.7%, and bilateral in 33.3%.

Because all treated patients met the pre-specified manometric threshold for intervention, the observed gradient values in this cohort should not be interpreted as defining a discriminatory cutoff for treatment selection. Lumbar puncture (LP) opening pressure decreased from 39.93 ± 10.99 cm H₂O before stenting to 18.23 ± 3.87 cm H₂O at the 1-month post-stenting follow-up visit; this pre- to 1-month post-stenting change is reported in the main Results text rather than in the baseline characteristics table. Expanded baseline characteristics available for standardized retrospective extraction, including BMI, selected comorbidities, symptom duration, prior treatment history, and baseline papilledema findings, are summarized in Table 1. 

**Table 1:** Available baseline sociodemographic, clinical, ophthalmologic, and physiologic characteristics of the study cohort (*N* = 30)

Characteristic	Value
Age, years, mean ± SD	39.0 ± 13.1
Age, years, median	38.5
Sex, n (%)	Female: 28 (93.3%); Male: 2 (6.7%)
BMI, kg/m², mean	31.6
BMI, kg/m², median	31.8
Hypertension, n (%)	12 (40.0%)
Diabetes mellitus, n (%)	6 (20.0%)
Duration of symptoms, months, mean	8.9
Duration of symptoms, months, median (range)	8.0 (2–20)
PT laterality, n (%)	Right: 15 (50.0%); Left: 5 (16.7%); Bilateral: 10 (33.3%)
Pre-stent trans-stenotic venous pressure gradient, mmHg, mean ± SD	7.0 ± 1.4
Pre-stent trans-stenotic venous pressure gradient, mmHg, median (range)	7.0 (4.5–10)
Pre-stent LP opening pressure, cm H₂O, mean ± SD	39.93 ± 10.99
Pre-stent LP opening pressure, cm H₂O, median (range)	38.5 (25–65)
Papilledema before stenting, n (%)	30 (100%)
Pre-stenting papilledema grade, mean	2.43
Grade 1 papilledema, n (%)	2 (6.7%)
Grade 2 papilledema, n (%)	15 (50.0%)
Grade 3 papilledema, n (%)	11 (36.7%)
Grade 4 papilledema, n (%)	2 (6.7%)
Prior acetazolamide therapy, n (%)	21 (70.0%)
Prior repeated lumbar puncture, n (%)	10 (33.3%)

Data are presented as mean ± SD, median (range), or n (%), as appropriate. *PT* pulsatile tinnitus, *LP* lumbar puncture, *BMI* body mass index

Papilledema improved markedly after venous sinus stenting. Before stenting, papilledema was documented in all patients (30/30, 100%). Grade 2 papilledema was the most common baseline finding (15/30, 50.0%), followed by grade 3 (11/30, 36.7%), grade 1 (2/30, 6.7%), and grade 4 (2/30, 6.7%). The mean papilledema grade decreased from 2.43 before stenting to 0.30 during follow-up. At follow-up, 21 patients (70.0%) had complete resolution of papilledema with normal optic disc appearance, while 9 patients (30.0%) had only mild residual grade 1 papilledema. No patient had persistent grade 2, grade 3, or grade 4 papilledema after stenting.

### Primary clinical outcome (immediate post-stenting)

Immediate complete resolution of PT occurred in 27 of 30 patients (90%). Three patients (10%) had persistent PT with partial improvement immediately after the procedure (Table 2).

**Table 2:** Clinical status of pulsatile tinnitus at baseline, immediate post-stenting, and up to 12 months after venous sinus stenting (*N* = 30)

Clinical status	Baseline (pre-stent), *n* (%)	Immediate post-stent, *n* (%)	Up to 12 months post-stent, *n* (%)
PT present (symptomatic)	30 (100)	3 (10)	0 (0)
PT absent (complete resolution)	0 (0)	27 (90)	30 (100)

Immediate post-stent” reflects the first documented outcome after the procedure; “Up to 12 months” reflects the latest documented follow-up status within the first year after the procedure and does not imply a uniform 12-month visit for all patients

### Procedural complications

No peri-procedural complications were reported in 27 of 30 patients (90.0%). Minor complications occurred in 3 patients (10.0%), including ecchymosis (i.e., localized subcutaneous bruising/bleeding, [at the vascular access site]) in 2 patients (6.7%) and epistaxis in 1 patient (3.3%). No major adverse events were documented.

### Hemodynamic and radiographic outcomes

Stenting was associated with significant hemodynamic and physiologic improvement. The mean trans-stenotic pressure gradient decreased from 7.0 ± 1.4 mmHg pre-stenting to 2.3 ± 1.1 mmHg post-stenting (*P* < 0.001). Similarly, mean LP opening pressure decreased from 39.93 ± 10.99 cm H₂O before stenting to 18.23 ± 3.87 cm H₂O at the 1-month post-stenting follow-up visit (*P* < 0.001). One-month post-stenting LP opening pressure measurements were available for all included patients (30/30).

The mean stenosis length was 9.2 ± 3.1 mm. On follow-up MRV and/or post-stenting angiographic assessment, neuroradiology review documented resolution of the treated stenotic segment with restored venous sinus caliber/patency and absence of residual flow-limiting stenosis in 30/30 patients (100%).

This assessment was based on neuroradiological review and a descriptive/semi-quantitative assessment of restored venous sinus patency, rather than on a retrospectively measured percentage reduction in stenosis.

**Fig. 1:**
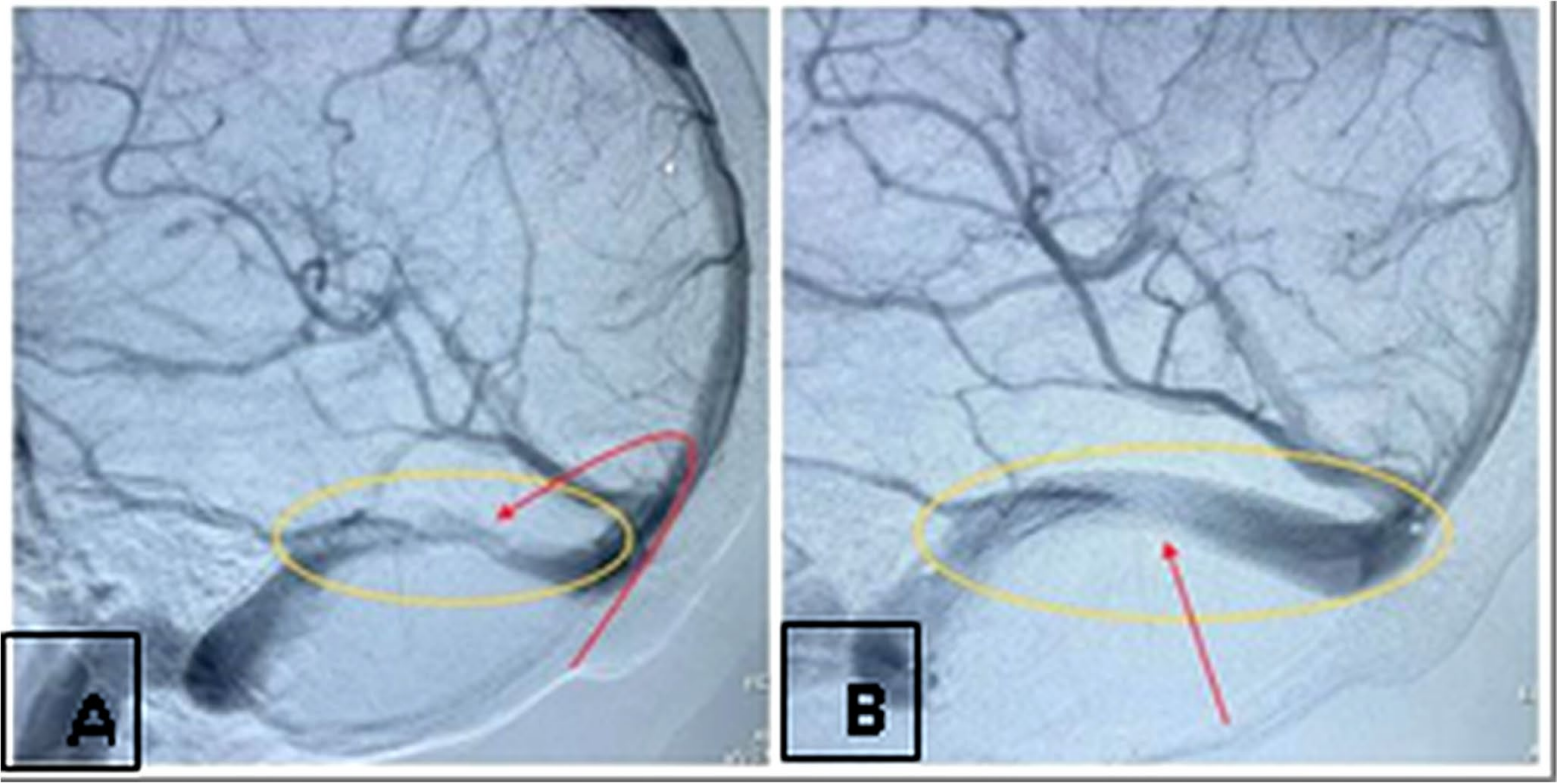
Representative case of right-sided pulsatile tinnitus treated with venous sinus stenting. The patient had a pre-stenting trans-stenotic pressure gradient of 10 mmHg, which decreased to 3 mmHg after stent deployment. LP opening pressure decreased from 37 cm H₂O before stenting to 21 cm H₂O during follow-up, and the patient experienced immediate complete resolution of pulsatile tinnitus. (A) Pre-stenting venography/DSA demonstrated hemodynamically significant transverse/sigmoid venous sinus stenosis corresponding to the symptomatic side. (B) Post-stenting venography/DSA showed improved sinus caliber and restoration of venous outflow across the treated segment, consistent with the procedural findings and post-stenting manometric improvement

Representative pre- and post-stenting angiographic findings from one illustrative case are shown in Fig. 1.

## Discussion

In this dual-center retrospective cohort of 30 patients with pulsatile tinnitus (PT) secondary to lateral venous sinus stenosis, venous sinus stenting was associated with high rates of symptom resolution, objective improvement in hemodynamic measures, and a low rate of minor complications. Clinically, complete resolution was achieved in 90% of patients immediately after stenting, and the remaining symptomatic patients improved to complete resolution by 12 months of follow-up. Follow-up MRV demonstrated resolution of the treated stenotic segment in all cases, and paired measurements demonstrated a significant reduction in the trans-stenotic pressure gradient. The 1-month change in LP opening pressure was interpreted as supportive early physiologic information rather than a stand-alone determinant of treatment response.

### Pathophysiologic rationale and procedural target

In venous pulsatile tinnitus related to transverse/sigmoid sinus stenosis, the proposed mechanism is turbulent venous flow across a hemodynamically significant narrowing, which may generate an audible pulse-synchronous sound. Venous sinus stenting is intended to restore venous outflow, improve sinus caliber, and reduce the trans-stenotic pressure gradient, thereby decreasing turbulence and improving symptoms.

In this study, a trans-stenotic pressure gradient threshold of ≥ 4 mmHg was used as a practical, pre-specified criterion to inform procedural selection, informed by the published venous sinus stenting literature. However, because all treated patients in this cohort exceeded that threshold and the observed gradient range was relatively narrow, the present results should not be interpreted as validating or refining a specific manometric cutoff for intervention. Rather, the pressure gradient was used as one component of an overall symptom–imaging–manometry framework, and the decision to proceed with stenting was based on the combined clinical, radiologic, and manometric profile rather than the pressure gradient value alone [11–13].

### Clinical outcomes

Our clinical results are consistent with previously published venous sinus stenting series in pulsatile tinnitus. The present study adds dual-center real-world data from a routine practice setting, with concurrent reporting of symptom response, trans-stenotic pressure-gradient reduction, 1-month change in LP opening pressure, radiologic resolution, papilledema improvement, and procedure-related complications. These findings should be interpreted in the context of the study’s retrospective, non-comparative design. Larger prospective studies with standardized baseline assessment, ophthalmologic documentation, and follow-up methodology remain necessary to refine selection criteria and predictors of response [11, 13].

The available literature on venous sinus stenting for pulsatile tinnitus remains heterogeneous in patient selection, outcome definitions, and follow-up methodologies. In our cohort, the elevated LP opening pressure profile suggests substantial overlap with raised intracranial pressure and possible IIH-spectrum physiology. However, because formal IIH diagnostic elements were not consistently documented in the retrospective records, we were unable to reliably classify all patients using a standardized IIH framework or to perform a robust subgroup analysis by IIH status. Accordingly, our findings should be interpreted as real-world outcomes in a venous PT cohort with marked overlap with raised-ICP, rather than as evidence from a strictly characterized non-IIH population [11, 13].

As a secondary early physiologic outcome, LP opening pressure decreased at 1 month after stenting in this cohort. Because LP opening pressure was measured using a standardized lateral-decubitus manometry protocol and was not measured in the sitting position, these data provide supportive physiologic information alongside the primary clinical, manometric, ophthalmologic, and imaging outcomes. The elevated baseline LP values were interpreted in the context of the cohort’s raised-intracranial-pressure phenotype, including documented papilledema in all patients, rather than as isolated measurements. However, LP opening pressure is not a continuous intracranial-pressure monitoring metric, and the present analysis does not provide a longitudinal pressure trajectory beyond the early 1-month reassessment.

### Recurrence and relation to IIH

In our cohort, persistent symptoms immediately after stenting and delayed symptom recurrence were uncommon during the available follow-up period. Similar favorable short- to intermediate-term symptom control has been reported in prior venous sinus stenting series in pulsatile tinnitus, including single-center cohorts of appropriately selected patients [9]. Larger pooled analyses suggest that recurrence or persistent symptoms may occur in a subset of patients after venous sinus stenting and that some patients may require longer surveillance and, rarely, repeat endovascular intervention [14]. Because our cohort was not designed as an IIH-only series and formal IIH status was not uniformly documented in the retrospective records, we were unable to perform a reliable subgroup analysis of recurrence by IIH status. Accordingly, our recurrence observations should be interpreted as real-world outcomes in a venous pulsatile tinnitus cohort rather than as IIH-specific recurrence estimates.

### Imaging, patient selection, and technique considerations

High-quality venous imaging and venous manometry remain central to patient selection for venous sinus stenting in pulsatile tinnitus. In clinical practice, cross-sectional imaging (e.g., MRV/CTV, with arterial imaging when indicated) helps identify a plausible venous source and exclude alternative vascular etiologies. At the same time, catheter venography with manometry confirms the hemodynamic significance of the stenotic segment before treatment. In our cohort, this symptom–imaging–manometry framework guided procedural selection and was used intra-procedurally to confirm technical success after stent deployment. This approach is consistent with prior venous sinus stenting series that emphasize structured selection criteria and objective hemodynamic assessment [9, 11].

### Limitations

This study has several limitations. First, the retrospective dual-center design and small sample size (*N* = 30) limit causal inference, reduce the precision of effect estimates, and restrict the ability to conduct robust multivariable analyses of predictors for symptom response, complications, or recurrence. The study should therefore be interpreted as an observational, real-world cohort study rather than a comparative or indication-defining analysis.

Second, although additional baseline and ophthalmologic variables were extracted after re-reviewing the records, including BMI, selected comorbidities, symptom duration, selected previous treatment history, and papilledema status, the retrospective design still limited the completeness and standardization of some clinically important variables. Detailed comorbidity severity, medication adherence, formal IIH diagnostic classification elements, and selected PT-related radiologic features, such as sigmoid sinus dehiscence/diverticulum, were not uniformly available for standardized analysis.

Third, LP opening pressure was reassessed at 1 month after stenting and was available for all included patients (30/30). However, the present analysis reports the change in LP from pre-stenting to 1-month post-stenting, rather than a longitudinal LP pressure trajectory across later follow-up intervals. Therefore, the LP findings should be interpreted as an early follow-up physiologic response rather than as a detailed time-dependent analysis of intracranial pressure changes after stenting.

Fourth, clinical follow-up was scheduled at 3, 6, and 12 months after stenting, and the latest documented clinical status within the first post-procedural year was used for outcome reporting. Nevertheless, because follow-up was based on routine clinical practice rather than a prospective research protocol, minor variability in follow-up documentation and timing may affect the interpretation of symptom durability, recurrence, and imaging outcomes.

Fifth, radiologic resolution was assessed descriptively through neuroradiology review based on restored venous sinus caliber/patency and absence of residual flow-limiting stenosis, rather than using a uniformly measured quantitative percentage reduction in stenosis. Finally, because all treated patients met the pre-specified minimum trans-stenotic pressure gradient threshold for intervention, the present dataset does not permit derivation of a more precise gradient-based selection cutoff.

### Practical implications and future directions

Our findings support venous sinus stenting as a potentially effective, low-complication treatment option for carefully selected patients with venous pulsatile tinnitus and documented venous sinus stenosis. However, the present study should not be interpreted as defining definitive treatment indications. Rather, its practical contribution lies in showing how a symptom–imaging–manometry framework was applied in routine dual-center practice, alongside reporting of symptom outcomes and objective physiologic improvement. At the same time, some clinically relevant elements, including formal IIH diagnostic classification, detailed comorbidity severity, medication adherence, and selected PT-related radiologic features, were not uniformly available in the retrospective records, limiting the precision with which selection factors can be inferred from this cohort.

Future work should focus on prospective registries, standardized baseline characterization, ophthalmologic assessment, harmonized outcome reporting, and comparative evaluation of selection frameworks across different venous sinus pathologies and treatment strategies.

Future prospective studies should incorporate standardized ophthalmologic assessment, including baseline and follow-up documentation of papilledema, along with structured manometric and imaging evaluation.

## Conclusion

Venous sinus stenting was associated in this cohort with high rates of pulsatile tinnitus resolution, significant reduction in the trans-stenotic pressure gradient, improvement in papilledema, radiologic resolution, and a low rate of minor complications; the 1-month change in LP opening pressure provided supportive early physiologic information. The principal contribution of the study is not the establishment of definitive treatment indications, but rather the reporting of dual-center real-world outcomes within a symptom–imaging–manometry framework used for procedural selection. These findings support the feasibility and potential effectiveness of this approach in routine practice, while also underscoring that the retrospective design and incomplete availability of some baseline clinical variables limit the extent to which treatment indications can be refined from the present dataset alone. Larger prospective studies with standardized selection criteria, ophthalmologic assessment, and longer follow-up are needed to define predictors of response and recurrence better and to inform patient selection.

### Ethical approval

The study protocol was reviewed and approved by the Research Ethics Committee, Faculty of Medicine, Port Said University, Egypt (ERN: MED (1/06/2025); Serial No. 296; NUS_003; approval date: June 2025). The study was conducted in accordance with the Declaration of Helsinki. Patient data were handled confidentially and analyzed in an anonymized form. Given the retrospective design, the ethics committee waived the requirement for informed consent.

### Study population

We included consecutive adult patients presenting with pulsatile tinnitus (PT) who underwent venous sinus stenting for radiologically confirmed transverse and/or sigmoid sinus stenosis. Clinical, radiologic, procedural, and follow-up data were retrieved from medical records.

### Eligibility criteria

#### Inclusion criteria

 [1] age ≥ 18 years; [2] radiologic evidence of transverse and/or sigmoid sinus stenosis; [3] documented pre-stenting clinical data, preprocedural noninvasive venous imaging, intraprocedural catheter venography/DSA with venous manometry, and post-stenting clinical and follow-up venous imaging data and [4] follow-up duration ≥ 3 months. For outcome reporting, follow-up status was summarized using the latest documented clinical assessment within the first 12 months after stenting. 

The cohort was not restricted to patients with a uniformly documented formal diagnosis of idiopathic intracranial hypertension (IIH). In this retrospective series, the elevated LP opening pressure profile suggests substantial overlap with raised intracranial pressure and possible IIH-spectrum physiology. However, formal IIH classification was not consistently recorded using a standardized diagnostic framework across all charts. Accordingly, we did not retrospectively assign definitive IIH status to all patients, nor did we perform IIH-specific subgroup analyses.

#### Exclusion criteria

Non-vascular tinnitus, incomplete records, or prior cranial venous stenting or surgery.

### Baseline assessment and data collection

The variables collected included age, sex, body mass index (BMI), laterality of tinnitus, duration of tinnitus, associated symptomatology, and other baseline clinical findings retrievable from the retrospective records. Funduscopic examination findings and papilledema grade were extracted from the available ophthalmologic/funduscopic documentation before and after stenting. Papilledema grade was summarized descriptively before stenting and during follow-up to characterize the raised-intracranial-pressure phenotype and anatomical response after restoration of venous outflow.

Baseline radiologic assessment included noninvasive venous imaging using MR venography (MRV) and/or CT venography (CTV) to identify transverse and/or sigmoid sinus stenosis and to exclude alternative structural or vascular causes when clinically indicated. All patients subsequently underwent intraprocedural catheter venography/digital subtraction angiography (DSA) with venous manometry before stent deployment. This invasive assessment was used to confirm the anatomic stenosis, assess venous outflow, and quantify the trans-stenotic pressure gradient across the treated segment.

### Symptom severity assessment

PT severity and functional impact were extracted from clinical documentation at baseline and follow-up visits. Patients with clinically meaningful symptom burden (at least moderate severity) were considered for intervention after confirming a venous etiology.

### Endovascular procedure

Experienced neurointerventional teams performed all procedures. Venous access was obtained via the femoral or basilic vein under local anesthesia. A catheter was advanced through the jugular venous system to the superior sagittal sinus for venography and manometry. A microcatheter was navigated across the stenotic segment to obtain pressure measurements. The trans-stenotic pressure gradient was determined by comparing pressures across the stenosis, including measurements at the confluence of sinuses (torcular Herophili) and the distal sigmoid sinus.

A hemodynamically significant pressure gradient was required to proceed with stenting. In this study, a trans-stenotic pressure gradient of ≥ 4 mmHg, measured between the torcular Herophili and the distal sigmoid sinus, was used as a practical pre-specified procedural selection threshold before treatment decisions were made. This threshold was not derived from the present dataset and was not a post hoc analytical cutoff. Rather, it was selected according to published venous sinus stenting literature and institutional practice, while acknowledging that pressure-gradient thresholds differ across studies and that no universally accepted cutoff has been established [11–13].

Prior to stent deployment, arterial angiography was performed via radial artery access in all patients as part of the pre-procedural vascular assessment to evaluate arterial anatomy and exclude concomitant arterial causes of pulsatile tinnitus (e.g., dural arteriovenous fistula, carotid/vertebral stenosis or dissection, or aneurysmal lesions), particularly when suggested by clinical findings or noninvasive imaging.

Stent deployment was subsequently performed under general anesthesia. A self-expanding nitinol carotid stent system (PRECISE PRO RX^®^, Cordis, Miami Lakes, FL, USA) was used in all cases and deployed across the stenotic transverse/sigmoid sinus segment.

Following stent deployment under general anesthesia, venography and repeat manometric measurements were performed to confirm improved venous outflow and a reduced trans-stenotic pressure gradient.

### Antiplatelet regimen

All procedures were elective. All patients received dual antiplatelet therapy with aspirin 325 mg and clopidogrel 75 mg for five days before the procedure. No emergency stenting procedures were performed, and no patient required deviation from the planned pre-procedural antiplatelet regimen. Dual antiplatelet therapy was continued for three months after stenting, followed by aspirin monotherapy for an additional eleven months.

### Outcome measures and follow-up

The **primary clinical outcome** was PT resolution (complete, partial, or no improvement) assessed immediately after the procedure and during follow-up.

**Secondary outcomes** included: [1] change in trans-stenotic venous pressure gradient; [2] change in lumbar puncture (LP) opening pressure (measured pre-stenting and reassessed during post-stenting clinical follow-up) [3] radiologic improvement on follow-up venous imaging; and [4] procedure-related complications and recurrence. Radiologic improvement was defined as follow-up venous imaging demonstrating resolution of the treated stenotic segment, with restoration of venous sinus caliber/patency and absence of residual flow-limiting stenosis. Follow-up imaging was assessed through neuroradiology review as part of routine clinical follow-up. Radiologic resolution was assessed descriptively based on restored venous sinus caliber/patency and absence of residual flow-limiting stenosis at the treated segment on follow-up MRV and/or post-stenting angiographic assessment. A formal quantitative percentage reduction in stenosis, such as a > 50% reduction criterion, was not consistently measured retrospectively and therefore was not used as an outcome criterion.

Follow-up assessments were documented at routine clinical visits, including early follow-up and up to 12 months. The “up to 12 months” time point represents the latest documented follow-up status within the first post-procedural year and does not necessarily indicate a uniform visit at exactly 12 months for all patients.

Lumbar puncture (LP) opening pressure was included as a secondary physiologic outcome because it provided an objective measure related to intracranial pressure physiology in this venous pulsatile tinnitus cohort. In the context of the elevated pre-stenting LP values observed in the cohort, these measurements likely reflect substantial overlap with raised intracranial pressure and possible IIH-spectrum physiology; however, formal diagnostic classification of IIH was not uniformly documented in the retrospective records.

LP opening pressure was recorded before stenting and reassessed at the 1-month post-stenting follow-up visit. LP opening pressure was measured using a standard spinal manometer with the patient in the lateral decubitus position before CSF withdrawal. The measurement was not performed in the sitting position. The 1-month LP opening pressure value was used as a secondary early physiologic outcome for the pre- to post-stenting comparison. LP opening pressure was interpreted alongside the clinical presentation, papilledema status, venous manometry, and imaging findings rather than as a stand-alone measure of treatment response. Clinical follow-up was subsequently scheduled at 3, 6, and 12 months after stenting, and the latest documented clinical status within the first post-procedural year was used for symptom and recurrence outcome reporting.

Baseline variables listed in Table 1 were limited to variables that were consistently retrievable from the retrospective records. After re-reviewing the records, additional baseline variables were extracted and summarized, including BMI, selected comorbidities, symptom duration, selected previous treatment history, and baseline papilledema grade. Detailed comorbidity severity, medication adherence, formal IIH diagnostic classification elements, and selected PT-related radiologic features such as sigmoid sinus dehiscence/diverticulum were not uniformly available for standardized analysis.

### Statistical analysis

Continuous variables are presented as mean ± standard deviation (SD), and categorical variables are presented as frequencies (percentages). Pre- and post-stenting paired measurements were compared using appropriate paired tests. Associations between baseline factors and clinical outcome were explored using appropriate univariable analyses. A two-sided P value < 0.05 was considered statistically significant. Analyses were performed using IBM SPSS Statistics (version 26; IBM Corp., Armonk, NY, USA).

## Supplementary Information

Below is the link to the electronic supplementary material.

Supplementary Material 1

## Data Availability

The datasets used and/or analyzed during the current study are available from the corresponding author on reasonable request.

## Declarations

### Clinical trial registration

Not applicable. This is a retrospective observational cohort study; no participants were prospectively assigned to an intervention for research purposes.
