# Supplementary material for: Venous sinus stenting for pulsatile tinnitus secondary to venous sinus stenosis: a dual-center retrospective cohort study
Source: Neurosurg Rev. 2026 Jul 24;49(1):493. doi: 10.1007/s10143-026-04412-9 (PMC13396035; doi:10.1007/s10143-026-04412-9)
Supplement: Supplementary file 1 — Supplementary Material 1 [file 10143_2026_4412_MOESM1_ESM.docx]

**STROBE Checklist (Complete) — Cohort Study (Filled)**

**Manuscript:** Venous Sinus Stenting for Pulsatile Tinnitus Secondary to Venous Sinus Stenosis
**Design:** Dual-center retrospective cohort study

| Item | Recommendation (STROBE) | Reported on (Section/Tables/Figures) | Status / Notes |
| --- | --- | --- | --- |
| 1(a) | Indicate the study’s design in the title or abstract. | Title: Abstract (Methods line) | Yes |
| 1(b) | Provide an informative and balanced abstract. | Abstract (Background/Methods/Results/Conclusion/Keywords) | Yes |
| 2 | Explain the scientific background and rationale. | Introduction (all paragraphs) | Yes |
| 3 | State specific objectives, including prespecified hypotheses. | Introduction (Aim paragraph) | Yes |
| 4 | Present key elements of the study design early in the paper. | Materials and Methods → Study design and setting | Yes |
| 5 | Describe the setting, locations, and relevant dates. | Materials and Methods → Study design and setting (Jan 2018–Dec 2024; both centers) | Yes |
| 6(a) | Eligibility criteria, sources/methods of selection, and methods of follow-up. | Materials and Methods → Study population; Eligibility criteria; Outcome measures and follow-up | Yes |
| 6(b) | For matched studies: matching criteria and numbers. | Not applicable | NA (not a matched study) |
| 7 | Define outcomes, exposures, predictors, confounders, effect modifiers; diagnostic criteria, if applicable. | Materials and Methods → Outcome measures and follow-up; Baseline assessment and data collection | Partial (predictors/confounders not specified; acceptable if not analyzed) |
| 8 | Data sources/measurement; comparability if >1 group. | Materials and Methods → Baseline assessment and data collection; Endovascular procedure | Yes |
| 9 | Describe efforts to address potential sources of bias. | Materials and Methods → Study population (consecutive cohort); Discussion → Limitations | Partial (bias mitigation not explicitly detailed; consider adding 1–2 sentences) |
| 10 | Explain how the study size was arrived at. | Materials and Methods → Study population (consecutive during period); Results → first paragraph (N=30) | Yes |
| 11 | Explain the handling of quantitative variables. | Materials and Methods → Statistical analysis | Yes |
| 12(a) | Describe all statistical methods, including confounding control. | Materials and Methods → Statistical analysis | Partial (no confounding control reported; OK if not performed) |
| 12(b) | Methods for subgroups/interactions. | Not applicable | NA (no subgroup/interactions) |
| 12(c) | Explain how missing data were addressed. | Not explicitly stated | Missing (add: analyses performed on available data; report missing per variable) |
| 12(d) | Explain how loss to follow-up was addressed. | Results → follow-up outcomes up to 12 months (Table 3) | Partial (loss to follow-up not explicitly reported) |
| 12(e) | Describe sensitivity analyses. | Not applicable | NA (no sensitivity analyses) |
| 13(a) | Numbers at each stage (eligible, included, analyzed). | Results → Study cohort and baseline characteristics; Tables 1–5 | Partial (screening/eligibility counts not shown) |
| 13(b) | Reasons for non-participation at each stage. | Not reported | NA/Not available in retrospective records (if true) |
| 13(c) | Use a flow diagram. | Not included | Optional (not required, but can be added) |
| 14(a) | Characteristics of participants and exposures/confounders. | Results → Table 1 | Yes |
| 14(b) | Indicate the number with missing data for each variable. | Not reported | Missing (recommended to add a missing-data note) |
| 14(c) | Summarize follow-up time. | Results → Follow-up outcomes (Table 3; up to 12 months) | Partial (mean/median follow-up time not summarized) |
| 15 | Report outcome events or summary measures over time. | Results → Tables 2–5 | Yes |
| 16(a) | Provide estimates and precision; specify any adjusted confounders. | Results → Tables 1 & 5 (means ± SD; P values) | Partial (no 95% CI; no adjusted estimates) |
| 16(b) | Report category boundaries when continuous variables are categorized. | Not applicable | NA |
| 16(c) | Translate relative risk into absolute risk if relevant. | Not applicable | NA |
| 17 | Other analyses (subgroups, interactions, sensitivity). | Not applicable | NA |
| 18 | Summarize key results in relation to the objectives. | Discussion → opening paragraph | Yes |
| 19 | Discuss limitations, incl. bias/imprecision (direction/magnitude). | Discussion → Limitations | Yes |
| 20 | Cautious overall interpretation considering evidence. | Discussion (all paragraphs) | Yes |
| 21 | Discuss generalisability (external validity). | Discussion → Practical implications/future directions | Yes |
| 22 | Funding source and role. | Declarations → Funding | Yes |
